# Waist-hip ratio related genetic loci are associated with risk of impaired fasting glucose in Chinese children: a case control study

**DOI:** 10.1186/s12986-018-0270-2

**Published:** 2018-05-03

**Authors:** Qi-Ying Song, Xiang-Rui Meng, Anke Hinney, Jie-Yun Song, Tao Huang, Jun Ma, Hai-Jun Wang

**Affiliations:** 1 Department of Maternal and Child Health, School of Public Health, Peking University, No.38 Xueyuan Road, Haidian District, Beijing, 100191 China; 2 Institute of Child and Adolescent Health, School of Public Health, Peking University, Beijing, 100191 China; 3 Center for Global Health Research, Usher Institute of Population and Health Sciences and Informatics, University of Edinburgh, Edinburgh, Scotland UK; 4 Department of Child and Adolescent Psychiatry, Psychosomatics and Psychotherapy, University Hospital Essen, University of Duisburg-Essen, Essen, Germany; 5 Department of Epidemiology and Biostatistics, School of Public Health, Peking University, Beijing, 100191 China

**Keywords:** Glucose, WHR, Gene, Polymorphism, Children

## Abstract

**Background:**

The meta-analyses of genome-wide association studies identified several waist-hip ratio (WHR) related loci in individuals of European ancestry. Since the pattern of fat distribution and the relationship between fat distribution and glucose metabolism disturbance in Chinese are different from those in Europeans, the present study aimed to explore the individual and cumulative effects of WHR-related loci on glycemic phenotypes in Chinese children.

**Methods:**

A total of 2030 children were recruited from two independent studies. Eleven single nucleotide polymorphisms (SNPs) were selected and genotyped using matrix-assisted laser desorption ionization time of flight mass spectrometry (MALDI-TOF MS). Logistic regression and linear regression model were used to examine the association of 11 SNPs and genetic risk score (GRS) with impaired fasting glucose (IFG) and fasting plasma glucose (FPG), respectively.

**Results:**

Three SNPs (rs6795735, rs984222 and rs1011731) were nominally associated with IFG (all *P* < 0.05). Each WHR-increasing (C) allele of rs6795735 (*ADAMTS9*) was associated with a 40.1% increased risk of IFG (OR = 1.401, 95% CI = 1.131–1.735, *P* = 0.002), which remained significant after Bonferroni correction. We observed no association of both weighted and unweighted GRS with FPG and IFG (all *P* > 0.05).

**Conclusions:**

We identified individual effects of rs6795735 (*ADAMTS9*), rs984222 (*TBX15-WARS2*), and rs1011731 (*DNM3-PIGC*) on glycemic phenotypes in Chinese children for the first time. The study suggests that genetic predisposition to central obesity is associated with impaired fasting glucose, providing more evidence for the pathogenesis of diabetes.

**Electronic supplementary material:**

The online version of this article (10.1186/s12986-018-0270-2) contains supplementary material, which is available to authorized users.

## Background

Obesity is associated with type 2 diabetes mellitus (T2D) [1, 2]. The prevalence of obesity and T2D has increased during the past few decades, particularly in China [3, 4]. As the metabolic consequences of increasing fat mass are disproportionately attributable to the extent of central adiposity, measures of central adiposity, such as waist-hip ratio (WHR) and waist circumference, predict the risk of serious adverse metabolic outcomes better than body mass index (BMI) [5]. Among the indices involved in central obesity, WHR is of particular interest, as a measure of body fat distribution, because it integrates the adverse metabolic risk associated with increasing waist circumference with the more protective role of gluteal fat deposition [6].

A large meta-analysis of genome-wide association studies (GWAS) reported 14 single nucleotide polymorphisms (SNPs) associated with WHR independent of BMI [6]. By analyzing the associations between these loci and metabolic traits, they identified one locus (at *ADAMTS9*) reaching nominal significance for fasting plasma glucose (FPG) and three loci (at *ADAMTS9*, *NISCH-STAB1* and *ITPR2-SSPN*) reaching nominal significance for T2D (all *P* < 0.05). Afterwards, some studies also examined the associations of 14 WHR-related loci with glucose-related metabolic traits in other populations. For example, Burgdorf et al. [7] found that WHR-increasing allele (G) of rs4846567 (*LYPLAL1/SLC30A10*) was nominally associated with decreased concentration of FPG (*P* = 0.036). Similarly, Van Vliet-Ostaptchouk et al. [8] observed that the WHR-increasing allele at *CPEB4* (rs6861681) was significantly associated with lower FPG after adjusting for WHR (*P* = 2.3 × 10^− 4^), but the genetic risk score (GRS) for WHR was not associated with glucose-related traits. Another study by Huang et al. [9] showed that the genetic predisposition to central obesity, estimated using GRS based on the 14 WHR loci, was linearly related to higher T2D risk (OR = 1.04, 95% CI = 1.01–1.07, *P* = 0.01). All of these studies were replicated among European adult populations. However, the effects of the 14 WHR-related loci on glycemic phenotypes have not been analyzed in non-European ethnic groups. What’s more, the effects in children, which might be different from adults, have not been evaluated.

Therefore, we conducted the present study in a case-control sample of Chinese children to explore the individual and cumulative effects of WHR-related loci on glycemic phenotypes. Since the pattern of fat distribution and the relationship between fat distribution and metabolic disturbance in Chinese might be different from those in Europeans [10–12], our study will thus provide more evidence for the effects of these loci on glycemic phenotypes in a population with different ethnicity and at a younger age.

## Methods

### Participants

We conducted a case-control study in a total of 2030 children from two independent study groups, including 705 obese cases (BMI ≥ the 95th BMI percentile for Chinese children) and 1325 non-obese controls (BMI ≤ the 95th BMI percentile) recruited from the urban regions of Beijing, China. The first group came from the study on adolescent lipids, insulin resistance and candidate genes (ALIR) [13–15]. The second study group was from the Comprehensive Prevention project for Overweight and Obese Adolescents (CPOOA) with physical exercise and healthy nutrition as instruments [14–16]. The study design, recruitment of participants, and data collection of the two study groups was very similar, except for the age differences (ALIR study: children aged 14–17 years old; CPOOA study: children aged 7–18 years old), which had been described in detail previously [14, 15].

Both the ALIR study and CPOOA study were approved by the Ethics Committee of Peking University Health Science Center. Written informed consent was provided by all participants and, in the case of minors, their parents. Studies were performed according to the Declaration of Helsinki.

### Anthropometric and glycemic measurements

Anthropometric measurements, including height, weight, waist and hip circumferences were conducted according to standard protocols [14, 15]. BMI was calculated as weight (kg)/height (m^2^). For children aged 7–18 years, we used uniform BMI percentile criteria, which were determined in a representative Chinese population [17], to screen the obese and non-obese participants. Children with an age- and sex-specific BMI ≥ 95th percentile were defined as obese; those with an age- and sex-specific BMI less than the 95th percentile were defined as non-obese. Individuals who were underweight, according to the Chinese national screening criteria for malnutrition of school-age children and adolescents [18], and those with any vital organs diseases, such as heart disease, liver diseases, pulmonary diseases and kidney disease, were excluded.

Blood samples were drawn after a 12 h overnight fast. FPG concentrations were analyzed by a biochemical auto-analyzer (Hitachi 7060, Tokyo, Japan). Individuals with a FPG ≥ 5.6 mmol/L were considered as impaired fasting glucose (IFG) [19].

### SNPs selection and genotyping

The GWAS meta-analysis reported 14 SNPs associated with WHR [6]. Power calculation was performed using Quanto software (University of Southern California, Los Angeles, CA). With the assumed effect size (OR = 0.14) and effect allele frequency ≥ 0.12, statistical power to detect a positive association would be ≥75%, given our sample size. Hence, 12 SNPs with the minor allele frequency ≥ 0.12 in the Chinese population (Hapmap database: http://hapmap.ncbi.nlm.nih.gov) were initially selected and genotyped for the present study. Since Hardy-Weinberg equilibrium (HWE) was violated (*P* < 0.001) for rs718314, it was excluded from subsequent data analyses, leaving 11 SNPs for our present study. Genomic DNAs of all children were extracted from blood leukocytes by the phenol-chloroform extraction. Genotyping of the 11 SNPs was conducted with a MassARRAY System (Sequenom, San Diego, CA). The genotyping process has been described in detail in our previous studies [20, 21]. The call rates of the 11 SNPs ranged from 98.9% to 99.9%. We also performed genotyping on randomly selected duplicated samples, and the concordance rates were 100%.

### Statistical analyses

The genotype data of the non-obese and obese groups were tested for deviation from HWE separately. In order to estimate the effect of population subdivision, *F*-statistics (*F*_ST_) was calculated [22, 23]. A *F*_*ST*_ value between 0 and 0.05 suggests little genetic differentiation; a value between 0.05 and 0.15, moderate differentiation; a value between 0.15 and 0.25, large differentiation; and values above 0.25, very large differentiation [24].

A logistic regression model was used to examine the independent effect of each SNP on risk of IFG under an additive model adjusted for study group, sex, age and age squared, without or with adjustment for age- and sex-specific WHR-Z scores. WHR-Z scores were calculated using the means and standard deviations of our study population. A general linear model was used to examine the independent effect of each SNP on the FPG under an additive model with adjustment for study group, sex, age and age squared, without or with adjustment for age- and sex-specific WHR-Z scores.

To explore cumulative effects of the 11 SNPs on risk of IFG and FPG, weighted and unweighted GRS was implemented. None of the 11 SNPs was found to be in linkage disequilibrium (LD) with each other at an r^2^ < 0.05, which was calculated in Han Chinese population with LDLink (https://analysistools.nci.nih.gov/LDlink/). Unweighted GRS was calculated by adding the number of effect alleles of 11 SNPs carried by each individual. Weighted GRS was calculated using reported effect sizes (β-coefficients) in the original study, with the following equation: weighted GRS = (β1× SNP1 + β2× SNP2 +. .. + βn × SNPn) × (total number of SNPs / sum of the β-coefficients). The genotypes of each SNP were coded as 0, 1 and 2 according to the number of effect alleles. Effect alleles in the current study were consistent with those in the original study. Logistic regression and linear regression were used to study the associations between GRS and risk of IFG and FPG, respectively.

Due to the reported sexual dimorphism of WHR [6], we also performed sex-specific analysis for the single SNP analyses and GRS association. A two-sided *P* value < 0.05 was considered as nominal significance. Adjustment was made for multiple testing using Bonferroni correction for 11 SNPs and 2 models (with and without adjusting for the WHR z-scores), i.e. resulting in 0.00227 (0.05 divided by 22) as statistical significance (two-sided). Statistical analyses were performed using SPSS 18.0 (SPSS Inc., Chicago, IL).

## Results

The general characteristics of the study populations are shown in Table 1. The difference in age and FPG between obese and non-obese groups was not significant (*P >* 0.05), while the differences in gender, BMI, and waist circumference between the two groups were significant (*P* < 0.05). The comparisons of individuals with and without IFG were shown in the Additional file 1: Table S1.

**Table 1:** General characteristics of the study populations

	Non-obese	Obese	*P*-value
Number	1325	705	–
Female (%)	591 (44.6)	221 (31.3)	< 0.001*
Age, years	12.93 ± 2.72	12.85 ± 2.59	0.497
BMI, kg/m^2^	21.53 ± 3.45	28.12 ± 3.94	< 0.001*
Waist circumference, cm	71.78 ± 9.91	88.12 ± 11.27	< 0.001*
Hip circumference, cm	88.75 ± 11.23	100.68 ± 11.15	< 0.001*
WHR	0.81 ± 0.06	0.88 ± 0.06	< 0.001*
FPG, mmol/L	4.93 ± 0.66	4.90 ± 0.75	0.332
IFG (%)	184 (13.9)	104 (14.8)	0.595

Data were provided as mean ± s.d. if not indicated otherwise. *BMI* body mass index, *FPG* fasting plasma glucose

*WHR* waist-hip ratio

*P < 0.05

Table 2 shows the genotype information of the 11 SNPs in 2030 Chinese children. HWE was fulfilled for all SNPs both among non-obese children and obese children (*P* > 0.05). *F*_ST_ values between the population of original study and our sample are also shown in Table 2. We identified 5 SNPs (rs1011731, rs10195252, rs6795735, rs1294421, rs1055144 in or near *DNM3-PIGC*, *GRB14*, *ADAMTS9*, *LY86*, *NFE2L3*, respectively) with moderate genetic differences between the original and our study groups (0.05 ≤ *F*_ST_ ≤ 0.15), while the remaining 6 SNPs had similar allele frequencies compared to the previous data [6] (*F*_ST_ ≤ 0.05).

**Table 2:** Genotyping information of 11 GWAS-derived SNPs for WHR in 2030 Chinese children

SNP	Chr.	Position^a^	Nearest gene	Allele		EAF	Call rate (%)	genotype frequencies (%, 11/12/22)			HWE *P*-value		EAF in Heid et al., 2010 (Ref. [6])	*F*_ST_
				Effect (1)	Other (2)			Total sample	non-IFG	IFG	non-obese	obese		
rs984222	1	119,305,366	*TBX15-WARS2*	G	C	0.61	99.8	37.7/46.9/15.4	39.2/45.8/15.0	28.9/53.7/17.4	0.964	0.404	0.64	< 0.01
rs1011731	1	170,613,171	*DNM3-PIGC*	G	A	0.13	99.9	1.7/21.9/76.4	1.6/21.0/77.4	2.8/26.7/70.5	0.588	0.938	0.43	0.11
rs4846567	1	217,817,340	*LYPLAL1*	G	T	0.69	99.9	46.9/43.9/9.2	47.4/43.7/8.8	43.6/45.3/11.1	0.124	0.819	0.72	< 0.01
rs10195252	2	165,221,337	*GRB14*	T	C	0.89	99.2	78.8/20.1/1.1	79.2/19.7/1.1	76.4/22.5/1.1	0.951	0.234	0.60	0.11
rs6795735	3	64,680,405	*ADAMTS9*	C	T	0.24	99.8	5.9/36.6/57.5	5.6/35.7/58.6	7.6/42.0/50.3	0.267	0.082	0.59	0.13
rs1294421	6	6,688,148	*LY86*	G	T	0.23	99.9	5.3/34.6/60.1	5.1/34.6/60.3	6.3/34.4/59.4	0.338	0.620	0.61	0.15
rs6905288	6	43,866,851	*VEGFA*	A	G	0.74	99.5	55.2/38.5/6.3	55.7/37.9/6.4	52.6/41.8/5.6	0.559	0.904	0.56	0.04
rs9491696	6	127,494,332	*RSPO3*	G	C	0.53	99.4	29.1/48.9/22.1	28.9/48.8/22.3	30.1/49.3/20.6	0.988	0.218	0.48	< 0.01
rs1055144	7	25,837,634	*NFE2L3*	T	C	0.44	99.5	19.6/48.7/31.7	19.2/48.7/32.1	22.0/48.4/29.6	0.553	0.950	0.21	0.06
rs1443512	12	52,628,951	*HOXC13*	A	C	0.20	98.9	4.2/30.7/65.0	4.1/30.5/65.4	4.9/32.0/63.0	0.792	0.124	0.24	< 0.01
rs4823006	22	27,781,671	*ZNRF3-KREMEN1*	A	G	0.49	99.4	23.9/49.4/26.7	23.0/50.2/26.8	29.4/44.4/26.2	0.910	0.442	0.57	0.01

*Chr* Chromosome, *EAF* Effect allele frequency, *F*_ST_*F*-statistics, *HWE* Hardy-Weinberg equilibrium, *IFG* impaired fasting glucose, *SNP* single nucleotide polymorphism

^a^Position: NCBI build 36.3 (NCBI, Bethesda, MD)

The associations between the SNPs and risk of IFG in 2030 Chinese children are shown in Table 3. Three SNPs were found to be associated with the risk of IFG independent of WHR. Each WHR-increasing (G) allele of rs984222 (*TBX15*-*WARS2*) was associated with a 24.9% reduced risk of IFG (OR = 0.751, 95% CI = 0.620–0.910, *P* = 0.004); each WHR-increasing (G) allele of rs1011731 (*DNM3*-*PIGC*) was associated with a 38.3% increased risk of IFG (OR = 1.383, 95% CI = 1.063–1.799, *P* = 0.016); each WHR-increasing (C) allele of rs6795735 (*ADAMTS9*) was associated with a 40.1% increased risk of IFG (OR = 1.401, 95% CI = 1.131–1.735, *P* = 0.002), with additional adjustment for age- and sex-specific WHR-Z scores. The associations of rs6795735 (*ADAMTS9*) with the risk of IFG remained significant after Bonferroni correction. However, we observed no association of both weighted and unweighted GRS with IFG (all *P* > 0.05).

**Table 3:** Associations of the 11 SNPs with the risk of IFG in 2030 Chinese children

SNP	Nearest gene	Unadjusted for age- and sex-specific WHR-Z scores		Adjusted for age- and sex-specific WHR-Z scores	
		OR (95% CI)	*P*-value	OR (95% CI)	*P*-value
rs984222	*TBX15-WARS2*	0.756 (0.624, 0.915)	0.004*	0.751 (0.620, 0.910)	0.004*
rs1011731	*DNM3-PIGC*	1.374 (1.058, 1.786)	0.017*	1.383 (1.063, 1.799)	0.016*
rs4846567	*LYPLAL1*	0.894 (0.731, 1.093)	0.276	0.895 (0.731, 1.095)	0.280
rs10195252	*GRB14*	0.925 (0.691, 1.239)	0.602	0.931 (0.695, 1.248)	0.633
rs6795735	*ADAMTS9*	1.400 (1.131, 1.734)	0.002*	1.401 (1.131, 1.735)	0.002*
rs1294421	*LY86*	1.054 (0.843, 1.316)	0.646	1.043 (0.835, 1.305)	0.709
rs6905288	*VEGFA*	0.964 (0.778, 1.195)	0.736	0.952 (0.768, 1.181)	0.657
rs9491696	*RSPO3*	1.107 (0.916, 1.336)	0.293	1.117 (0.925, 1.349)	0.252
rs1055144	*NFE2L3*	1.118 (0.926, 1.349)	0.245	1.120 (0.927, 1.352)	0.240
rs1443512	*HOXC13*	1.135 (0.900, 1.431)	0.284	1.121 (0.888, 1.414)	0.337
rs4823006	*ZNRF3-KREMEN1*	1.175 (0.973, 1.419)	0.093	1.170 (0.969, 1.413)	0.103
Unweighted GRS	*–*	1.059(0.989, 1.134)	0.099	1.057 (0.987, 1.132)	0.111
Weighted GRS	*–*	1.042 (0.975, 1.114)	0.227	1.041 (0.973, 1.113)	0.245

Logistic regression was performed to examine the independent and cumulative effects of each SNP on risk of IFG under an additive model adjusted for study group, sex, age and age squared, without or with adjustment for age- and sex-specific WHR-Z scores

*CI* confidence interval, *GRS* genetic risk score, *IFG* impaired fast glucose (fasting plasma glucose ≥5.6 mmol/L), *OR* odds ratio; *SNP* single nucleotide polymorphism

*Two-sided *P* < 0.05

The associations between the SNPs and FPG in 2030 children are shown in Table 4. A nominally significant association was observed between rs984222 (*TBX15-WARS2*) and FPG. After additional adjustment for age- and sex-specific WHR-Z scores, each WHR-increasing (G) allele of rs984222 was associated with 0.032 mmol/L decrease in FPG (β = − 0.032, S.E. = 0.016, nominal *P* = 0.045). Both weighted and unweighted GRS was not associated with FPG concentrations, adjusted for study group, sex, age, age squared, with and without age- and sex-specific WHR-Z scores (all *P* > 0.05).

**Table 4:** Associations of the 11 SNPs with FPG in 2030 Chinese children

SNP	Nearest gene	Unadjusted for age- and sex-specific WHR-Z scores		Adjusted for age- and sex-specific WHR-Z scores	
		β (S.E.)	*P*-value	β (S.E.)	*P*-value
rs984222	*TBX15-WARS2*	−0.033 (0.016)	0.043*	− 0.032 (0.016)	0.045*
rs1011731	*DNM3-PIGC*	0.035 (0.024)	0.137	0.031 (0.024)	0.192
rs4846567	*LYPLAL1*	−0.015 (0.017)	0.390	−0.013 (0.017)	0.455
rs10195252	*GRB14*	0.010 (0.026)	0.704	0.011 (0.025)	0.671
rs6795735	*ADAMTS9*	0.033 (0.018)	0.078	0.032 (0.018)	0.083
rs1294421	*LY86*	0.010 (0.019)	0.597	0.011 (0.019)	0.565
rs6905288	*VEGFA*	−0.011 (0.018)	0.553	−0.011 (0.018)	0.545
rs9491696	*RSPO3*	0.020 (0.016)	0.196	0.021 (0.016)	0.174
rs1055144	*NFE2L3*	0.018 (0.016)	0.253	0.021 (0.016)	0.181
rs1443512	*HOXC13*	0.013 (0.020)	0.524	0.012 (0.020)	0.543
rs4823006	*ZNRF3-KREMEN1*	−0.007 (0.016)	0.677	−0.005 (0.016)	0.751
Unweighted GRS	*–*	0.004 (0.006)	0.464	0.005 (0.006)	0.391
Weighted GRS	*–*	0.004 (0.006)	0.502	0.004 (0.006)	0.418

Linear regression was performed to examine the independent and cumulative effects of each SNP on FPG under an additive model adjusted for study group, sex, age and age squared, without or with adjustment for age- and sex-specific WHR-Z scores

*FPG* fasting plasma glucose, *GRS*genetic risk score, *SNP* single nucleotide polymorphism, *S.E* standard error, *WHR* waist-hip ratio

*Two-sided *P* < 0.05

Except a nominally significant association between rs10195252 (*GRB14*) and WHR (*P =* 0.04), we did not observe associations for the remaining 10 SNPs and WHR in 2030 children (all *P* > 0.05; Additional file 1: Table S2). In addition, significant association of these SNPs with BMI was also not detected (all *P* > 0.10; Additional file 1: Table S2).

### Sex-stratified analyses

Further stratified analysis for sex showed that rs984222 (*TBX15-WARS2*) was nominally associated with IFG only in boys, while rs6795735 (*ADAMTS9*) and rs4846567 (*LYPLAL1*) were nominally associated with IFG only in girls (all *P* < 0.05, Additional file 1: Table S3). However, only rs6795735 (*ADAMTS9*) showed marked differences in sex-specific effect size (*P* = 0.001, Additional file 1: Table S3). The effect of weighted and unweighted GRS showed no sexual dimorphism in Chinese children (all *P* > 0.05, Additional file 1: Table S3).

## Discussion

To our knowledge, this is the first study investigating association of the 11 GWAS derived WHR loci with FPG and risk of IFG in Chinese children. We identified three SNPs (rs6795735, rs984222 and rs1011731) showed individual effect on glycemic phenotypes. After Bonferroni correction, the individual effect of rs6795735 (*ADAMTS9*) on the risk of IFG remained significant.

*ADAMTS9* encodes a member of the ADAMTS (a disintegrin and metalloproteinase with thrombospondin motifs) protein family, implicating in the cleavage of proteoglycans, the control of organ shape during development, and the inhibition of angiogenesis [25]. *ADAMTS9* was found to be involved in insulin signaling, which might have direct impact on glycemic phenotypes [6]. The SNP rs6795735 located in intron *ADAMTS9* was found to be associated with a 40.1% increased risk of IFG after additional adjustment for age- and sex-specific WHR-Z scores in our study (*P* = 0.002). This SNP was originally found to be associated with WHR in the GWAS [6]. The original study also identified the SNP to be associated with high density lipoprotein cholesterol (Z score = − 2.486, *P* = 0.013), fasting glucose (Z score = 2.031, *P* = 0.042) and the risk of T2D (OR = 1.124, *P* = 0.002). Later in 2012, another GWAS conducted by the DIAbetes Genetics Replication and Meta-analysis (DIAGRAM) Consortium reported that rs6795735 was significantly associated with T2D [26]. Afterwards, three studies explored the associations between rs6795735 and fat distribution or metabolic phenotypes, but did not report significant associations [7, 8, 27]. Our study is the first to validate the effect of rs6795735 on glycemic phenotypes in Chinese children. Our results suggest possible ethnic differences for effect of rs6795735 on glycemic phenotypes, demonstrating the value of conducting genetic epidemiology studies in populations with different ethnicity. However, more large-scaled association studies and functional studies are needed.

SNP rs984222 located near *TBX15* and *WARS2* was associated with WHR in the original study [6]. Each G allele increased the WHR by 0.034 on average (*P* = 8.69 × 10^− 25^). However, no association between rs984222 and metabolic traits (including glycaemic traits and blood lipids) was identified [6]. Subsequently, Burgdorf et al. [7] and Van Vliet-Ostaptchouk et al. [8] studied the effect of rs984222 on glycaemic traits in European populations. Hotta et al. [27] studied its impact on body fat distribution in Japanese. However, none of them found evidence supporting its association with these metabolic phenotypes. Therefore, the present study is the first to identify associations between rs984222 and glycemic phenotypes, independent of WHR. The inconsistent results may be explained by population differences. We conducted our study in children and adolescents, whose developmental and metabolic processes might have different characteristics compared with that of adults. Additionally, different ethnic backgrounds between Chinese and European population may partly account for the inconsistent effect of rs984222 on glycaemic traits.

The association of rs984222 and glycemic phenotypes could be explained by the evidence from functional studies of *TBX15* gene. *TBX15* gene encodes a transcription factor of a phylogenetically conserved family that regulates a variety of developmental processes. Tbx15 involved in craniofacial and limb development in the mouse [28, 29], and plays an important role in adipocyte differentiation and function [30]. Gesta et al. [31] found that Tbx15 is highly differentially expressed between visceral and subcutaneous fat in both humans and rodents. Differential expression of Tbx15 between fat depots plays an important role in the interdepot differences in adipocyte differentiation, triglyceride accumulation, and mitochondrial function, which may contribute to the risk of diabetes and metabolic diseases [30].

Moreover, our study found that the WHR-decreasing allele of rs984222 was significantly associated with increased risk of IFG. This finding is somehow paradoxical. Similarly, van Vliet-Ostaptchouk et al. [8] found that the WHR-increasing allele at *CPEB4* (rs6861681) was associated with lower fasting glucose after adjusting for WHR. The paradoxical associations have also been reported in other studies [7, 32, 33], which may be explained by the genetic pleiotropism, that is, some proteins play different roles in independent biological pathways [8]. Studies had shown that some obese individuals remain metabolically healthy despite having excessive accumulation of body fat, while a number of normal-weight individuals are metabolically unhealthy [34, 35]. The mechanism causing the inconsistent results needs to be further clarified by functional studies and comprehensively assessments of genetic associations with BMI, WHR, glycemic phenotypes and T2D risk.

Dynamin 3 (DNM3) is a member of the dynamin family of enzymes. Dominant-negative mutant in transfected dynamin enzymes could promote GLUT6 and GLUT8 to be accumulated on the surface of rat adipose cells [36]. *PIGC* encodes a subunit of the enzyme involving in lipid biosynthesis [6]. In the present study, rs1011731 (*DNM3-PIGC*) was nominally associated with IFG, and the relationship was not significant after Bonferroni correction. However, in the original GWAS, rs1011731 was reported to be associated with only WHR, while not associated with FPG and T2D [6]. The following replication studies didn’t found its association with glycaemic traits, either [7, 8]. More studies are required to investigate the association between rs1011731 and glycaemic traits, especially in East Asian population.

When analyses were conducted in a sex-stratified manner, we observed that the effect of rs6795735 (*ADAMTS9*) on IFG showed sexual dimorphism in Chinese children. In the original GWAS meta-analysis, seven of the 14 WHR-associated loci exhibited significant sexual dimorphism. And effect sizes were numerically greater in women than in men. Consistent with the original study, we also found that rs6795735 was associated with IFG only in girls, with effect sizes numerically greater in girls than in boys. Sex-specific genetic effects have long been acknowledged. However, the underlying molecular mechanism remains unclear. Some researches believed that the heterogeneity might due to the differences between boys and girls in body composition, patterns of weight gain, hormone biology, and the susceptibility to certain genetic and environmental factors [37]. More studies are required before a clear understanding of the underlying molecular mechanisms.

It was known that WHR is a measure of central obesity and associated with diabetes and prediabetes (including impaired glucose tolerance (IGT) and IFG) [6, 38]. Subjects with IFG have a prominent deficiency in insulin secretion, suggesting an association with insufficient pancreatic B-cell function [39]. We studied the correlations between WHR-related loci found by previous GWAS and WHR, BMI, or glycemic phenotypes (IFG and FPG). Although we failed to confirm the associations between these loci and WHR or BMI, we identified individual effects of the 3 SNPs (rs984222, rs6795735 and rs1011731) on glycemic phenotypes. The different findings between our study and previous studies may be partly explained by the fact that the pattern of fat distribution in Chinese differs from those in Europeans, i.e. Chinese individuals have excessive body fat at normal BMI or WHR [10–12]. In addition, we supposed that genetic predisposition to central obesity might be associated with risk of impaired pancreatic islet function, and hypothesized the effect of these loci on glycemic phenotypes might be relevant at earlier stage than that on WHR in our children sample. It had been shown that both insulin resistance and visceral fat deposition (especially liver and epicardial fat) could be the trigger of the metabolic syndrome, which may cause glycemic alteration ahead of measurable WHR changes [40].

One highlight of the present study was that our study sample was children. Many common complex diseases observed in adults have their developmental origin in childhood [41, 42]. Therefore, it is of great significance to understand how the WHR variants derived from adults operate in children and if they confer risk for childhood obesity and other obesity-related diseases.

The present study had limitations. Firstly, we selected several WHR related genetic loci which were recently identified by the GWAS by Heid and colleagues [6]. There were other WHR related loci reported since the publication of Heid et al. [43–46], and the addition of more WHR variants to the GRS models would make our results more accurate. However, we think this study could help to provide evidences for the effects of these variants identified by Heid and colleagues. Secondly, the sample size was limited. However, the power of rs6795735, which showed significant association with IFG after adjustment for Bonferroni correction, was 92%, suggesting that the associations might not be false-positive.

## Conclusions

In conclusion, we found individual effects of rs6795735 (*ADAMTS9*), rs984222 (*TBX15-WARS2*), and rs1011731 (*DNM3-PIGC*) on glycemic phenotypes. These associations were independent of WHR. Our findings indicated that genetic susceptibility to central obesity might be associated with risk of impaired pancreatic islet function, providing more evidence for the pathogenesis of diabetes.

## Additional file

Additional file 1: **Table S1.** General characteristics of study subjects with and without IFG, **Table S2**. Associations of 11 SNPs with WHR and BMI in 2030 Chinese children, **Table S3** Sex-dependent association of 11 SNPs and GRS with IFG in 2030 Chinese children. (DOCX 25 kb)

## Abbreviations

*ADAMTS9*: A disintegrin-like and metalloproteinase with thrombospondin type 1 motif 9 gene
ALIR: Adolescent lipids, insulin resistance and candidate genes
BMI: Body mass index
CPOOA: Comprehensive Prevention project for overweight and Obese Adolescents
*DNM3*: Dynamin 3
FPG: Fasting plasma glucose
*F*_ST_: *F*-statistics
GRS: Genetic risk score
GWAS: Genome-wide association studies
HWE: Hardy-Wei nberg equilibrium
IFG: Impaired fasting glucose
IGT: Impaired glucose tolerance
LD: Linkage disequilibrium
PCR: Polymerase chain reaction
*PIGC*: Phosphatidylinositol glycan anchor biosynthesis, class C
SCICO: School-based Comprehensive Intervention on Childhood Obesity
SNP: Single nucleotide polymorphism
T2D: Type 2 diabetes.
*TBX15*: T-box 15
*WARS2*: Tryptophanyl-tRNA synthetase 2
WHR: Waist-hip ratio
