# Supplementary material for: Waist-hip ratio related genetic loci are associated with risk of impaired fasting glucose in Chinese children: a case control study
Source: Nutr Metab (Lond). 2018 May 3;15:34. doi: 10.1186/s12986-018-0270-2 (PMC5934898; doi:10.1186/s12986-018-0270-2)
Supplement: Supplementary file 1 — Table S1. General characteristics of study subjects with and without IFG, Table S2. Associations of 11 SNPs with WHR and BMI in 2030 Chinese children, Table S3 Sex-dependent association of 11 SNPs and GRS with IFG in 2030 Chinese children. (DOCX 25 kb) [file 12986_2018_270_MOESM1_ESM.docx]

**Table S1** General characteristics of study subjects with and without IFG

|  | Non-IFG | IFG | *P*-value |
| --- | --- | --- | --- |
| Number | 1742 | 288 | - |
| Female (%) | 718 (41.2) | 94 (32.6) | 0.006* |
| Age, years | 13.09±2.62 | 11.79±2.73 | <0.001* |
| BMI, kg/m^2^ | 23.96±4.81 | 22.98±4.61 | 0.001* |
| Waist circumference, cm | 77.72±13.02 | 75.73±12.66 | 0.016* |
| Hip circumference, cm | 93.45±12.55 | 89.38±12 | <0.001* |
| WHR | 0.83±0.07 | 0.85±0.07 | <0.001* |
| FPG, mmol/L | 4.77±0.61 | 5.86±0.31 | <0.001* |

Data were provided as mean± s.d. if not indicated otherwise. BMI, body mass index; WHR,waist-hip ratio; FPG, fasting plasma glucose. **P*<0.05.

**Table S2.** Associations of 11 SNPs with WHR and BMI in 2030 Chinese children

| SNP | Nearest gene | WHR | |  | BMI | |
| --- | --- | --- | --- | --- | --- | --- |
|  |  | β (S.E.) | *P*-value |  | β (S.E.) | *P*-value |
| rs984222 | *TBX15-WARS2* | 0.002 (0.002) | 0.381 |  | -0.020 (0.125) | 0.872 |
| rs1011731 | *DNM3-PIGC* | -0.001 (0.003) | 0.743 |  | -0.210 (0.184) | 0.252 |
| rs4846567 | *LYPLAL1* | 0.003 (0.002) | 0.122 |  | -0.001 (0.134) | 0.991 |
| rs10195252 | *GRB14* | -0.006 (0.003) | 0.040* |  | -0.280 (0.196) | 0.154 |
| rs6795735 | *ADAMTS9* | -0.001 (0.002) | 0.596 |  | -0.045 (0.143) | 0.751 |
| rs1294421 | *LY86* | 0.004 (0.002) | 0.091 |  | 0.133 (0.146) | 0.364 |
| rs6905288 | *VEGFA* | 0.003 (0.002) | 0.155 |  | 0.039 (0.142) | 0.785 |
| rs9491696 | *RSPO3* | -0.002 (0.002) | 0.229 |  | -0.161 (0.122) | 0.188 |
| rs1055144 | *NFE2L3* | -0.001 (0.002) | 0.576 |  | -0.123 (0.123) | 0.318 |
| rs1443512 | *HOXC13* | 0.000 (0.002) | 0.828 |  | -0.197 (0.153) | 0.199 |
| rs4823006 | *ZNRF3-KREMEN1* | -0.001 (0.002) | 0.693 |  | -0.105 (0.122) | 0.390 |
| GRS | *-* | 0.000 (0.001) | 0.734 |  | -0.066 (0.043) | 0.121 |

Linear regression was performed to examine the independent and cumulative effects of each SNP on WHR under an additive model adjusted for study group, sex, age and age squared. BMI, body mass index; GRS, genetic risk score; S.E., standard error; SNP, single nucleotide polymorphism; WHR, waist-hip ratio. *Two-sided *P*<0.05.

**Table S3** Sex-dependent association of 11 SNPs and GRS with IFG in 2030 Chinese children

| SNP | Nearest gene | Boys | |  | Girls | |  | Sex  difference |
| --- | --- | --- | --- | --- | --- | --- | --- | --- |
|  |  | OR (95% CI) | *P*-value |  | OR (95% CI) | *P*-value |  | *P*-value |
| rs984222 | *TBX15-WARS2* | 0.737 (0.581, 0.935) | 0.012 |  | 0.782 (0.563, 1.087) | 0.144 |  | 0.008 |
| rs1011731 | *DNM3-PIGC* | 1.386 (0.990, 1.939) | 0.057 |  | 1.359 (0.883, 2.091) | 0.163 |  | 0.024 |
| rs4846567 | *LYPLAL1* | 1.019 (0.793, 1.310) | 0.881 |  | 0.697 (0.494, 0.984) | 0.040 |  | 0.103 |
| rs10195252 | *GRB14* | 0.852 (0.593, 1.226) | 0.389 |  | 1.070 (0.643, 1.779) | 0.795 |  | 0.864 |
| rs6795735 | *ADAMTS9* | 1.289 (0.981, 1.693) | 0.068 |  | 1.610 (1.139, 2.276) | 0.007 |  | 0.001 |
| rs1294421 | *LY86* | 1.180 (0.898, 1.552) | 0.235 |  | 0.815 (0.545, 1.218) | 0.318 |  | 0.858 |
| rs6905288 | *VEGFA* | 1.163 (0.849, 1.593) | 0.346 |  | 0.927 (0.643, 1.337) | 0.685 |  | 0.621 |
| rs9491696 | *RSPO3* | 1.099 (0.866, 1.393) | 0.438 |  | 1.163 (0.849, 1.593) | 0.346 |  | 0.240 |
| rs1055144 | *NFE2L3* | 1.162 (0.917, 1.473) | 0.214 |  | 1.066 (0.778, 1.460) | 0.692 |  | 0.345 |
| rs1443512 | *HOXC13* | 1.247 (0.931, 1.671) | 0.138 |  | 0.960 (0.648, 1.423) | 0.839 |  | 0.600 |
| rs4823006 | *ZNRF3-KREMEN1* | 1.147 (0.907, 1.451) | 0.252 |  | 1.220 (0.887, 1.677) | 0.221 |  | 0.101 |
| Unweighted GRS | *-* | 1.080 (0.992, 1.176) | 0.076 |  | 1.024 (0.913, 1.150) | 0.683 |  | 0.217 |
| Weighted GRS | *-* | 1.063 (0.978, 1.156) | 0.152 |  | 1.009 (0.902, 1.129) | 0.877 |  | 0.409 |

Logistic regression was performed for boys and girls separately to examine the independent and cumulative effects of each SNP on risk of IFG under an additive model adjusted for study group, age, age squared and age- and sex-specific WHR-Z scores. SNPs with *P* for sex difference <0.0045 (0.05/11) were considered to show a significant sex difference. SNP, single nucleotide polymorphism; IFG, impaired fast glucose (fasting plasma glucose ≥5.6 mmol/L); OR, odds ratio; CI, confidence interval; GRS, genetic risk score.
